# Characterization of CD8^+^ T Cell Differentiation following SIVΔnef Vaccination by Transcription Factor Expression Profiling

**DOI:** 10.1371/journal.ppat.1004740

**Published:** 2015-03-13

**Authors:** James M. Billingsley, Premeela A. Rajakumar, Michelle A. Connole, Nadine C. Salisch, Sama Adnan, Yury V. Kuzmichev, Henoch S. Hong, R. Keith Reeves, Hyung-joo Kang, Wenjun Li, Qingsheng Li, Ashley T. Haase, R. Paul Johnson

**Affiliations:** 1 Division of Immunology, New England Primate Research Center, Harvard Medical School, Southborough, Massachusetts, United States of America; 2 Yerkes National Primate Research Center, Emory University, Atlanta, Georgia, United States of America; 3 Crucell Holland BV, Leiden, The Netherlands; 4 Center for Virology and Vaccine Research, Beth Israel Deaconess Medical Center, Boston, Massachusetts, United States of America; 5 Division of Preventive and Behavioral Medicine, University of Massachusetts Medical Center, Worcester, Massachusetts, United States of America; 6 Nebraska Center for Virology and School of Biological Sciences, University of Nebraska-Lincoln, Lincoln, Nebraska, United States of America; 7 University of Minnesota, Microbiology Department, Minneapolis, Minnesota, United States of America; 8 Ragon Institute of MGH, MIT, and Harvard, Cambridge, Massachusetts, United States of America; Vaccine Research Center, UNITED STATES

## Abstract

The onset of protective immunity against pathogenic SIV challenge in SIVΔnef-vaccinated macaques is delayed for 15-20 weeks, a process that is related to qualitative changes in CD8^+^ T cell responses induced by SIVΔnef. As a novel approach to characterize cell differentiation following vaccination, we used multi-target qPCR to measure transcription factor expression in naïve and memory subsets of CD8+^+^ T cells, and in SIV-specific CD8^+^ T cells obtained from SIVΔnef-vaccinated or wild type SIVmac239-infected macaques. Unsupervised clustering of expression profiles organized naïve and memory CD8^+^ T cells into groups concordant with cell surface phenotype. Transcription factor expression patterns in SIV-specific CD8^+^ T cells in SIVΔnef-vaccinated animals were distinct from those observed in purified CD8^+^ T cell subsets obtained from naïve animals, and were intermediate to expression profiles of purified central memory and effector memory T cells. Expression of transcription factors elicited by SIVΔnef vaccination also varied over time: cells obtained at later time points, temporally associated with greater protection, appeared more central-memory like than cells obtained at earlier time points, which appeared more effector memory-like. Expression of transcription factors associated with effector differentiation, such as *ID2* and *RUNX3*, were decreased over time, while expression of transcription factors associated with quiescence or memory differentiation, such as *TCF7*, *BCOR* and *EOMES*, increased. CD8^+^ T cells specific for a more conserved epitope expressed higher levels of *TBX21* and *BATF*, and appeared more effector-like than cells specific for an escaped epitope, consistent with continued activation by replicating vaccine virus. These data suggest transcription factor expression profiling is a novel method that can provide additional data complementary to the analysis of memory cell differentiation based on classical phenotypic markers. Additionally, these data support the hypothesis that ongoing stimulation by SIVΔnef promotes a distinct protective balance of CD8^+^ T cell differentiation and activation states.

## Author Summary

The live attenuated vaccine SIVΔnef can induce robust CD8^+^ T cell- mediated protection against infection with pathogenic SIV in macaques. Thus, there is substantial interest in characterizing these immune responses to inform HIV vaccine design. Animals challenged at 15–20 weeks post vaccination exhibit robust protection, whereas animals challenged at 5 weeks post-vaccination manifest little protection. Since the frequency of SIV-specific T cells decreases from week 5 to week 20, it is likely that the quality of the response to challenge changes as virus-specific cells differentiate. We applied a novel approach of transcription factor expression profiling to characterize the differences in SIV-specific cell function and phenotype at more protected and less protected time points. Using unsupervised clustering methods informed by expression profiles assessed in purified CD8^+^ T cell subsets, we show that SIV-specific cells display expression profiles different than any purified CD8^+^ T cell subset, and intermediate to sorted effector memory and central memory subsets. SIV-specific cells overall appear more effector memory-like at week 5 post-vaccination, and more central memory-like at week 20 post-vaccination. Distinct profiles of CD8^+^ T cells specific for different SIV epitopes having different immune escape kinetics suggests maturation is regulated by ongoing low-level replication of vaccine virus.

## Introduction

Vaccination of rhesus macaques with SIVΔnef can induce robust immune responses and can protect the majority of vaccinated animals from challenge with wild-type SIV virus strains [1–3]. To date, SIVΔnef is the most efficacious of all vaccine strategies analyzed in the macaque model. Although safety concerns preclude the use of attenuated HIV as a human vaccine [4,5], understanding the biological basis for immune protection conferred by SIVΔnef may provide information important for the design of safe and efficacious HIV vaccines. Therefore, substantial efforts have been made to identify correlates of immune protection induced by SIVΔnef over the last two decades.

Correlates of immune protection induced by SIVΔnef and related attenuated SIV vaccines identified to date include both cellular and humoral adaptive immune responses [3,6–10]. CD8^+^ T cell responses in particular appear to be critical for SIVΔnef-mediated protection. SIVΔnef can induce robust CD8^+^ CTL responses and protection can occur in the absence of neutralizing antibody responses [3,7]. Additionally, CD8^+^ cell depletion following vaccination with attenuated SIV vaccines results in impaired control of challenge virus [6,11]. Although the vaccine virus is rapidly cleared to levels in plasma that are at or below detection following vaccination in the majority of animals [1], virus continues to replicate at low levels [12,13]. Substantial evidence suggests that the replicative capacity of SIVΔnef, and the ability to provide persistent low-level antigenic stimulation, may mediate the high efficacy of protection [3,12–14]. A comparison of SIVΔnef with more-attenuated SIV virus strains found a positive correlation of the magnitude of lymph-node resident SIV-specific T cells to protection from intravenous challenge [13]. However, SIVΔnef does not induce greater numbers of SIV-specific CD8^+^ T cells than other vaccine approaches that do not induce protection [15–17]. Additionally, the frequency of SIVΔnef-induced SIV-specific CD8^+^ T cells located in lymphoid and genital tissues does not correlate with the maturation of protection in this model [12]. The frequency or magnitude, therefore, of virus-specific precursors in blood and tissues may be less important for immune control than the composition of CD8^+^ T cell memory phenotypes induced by SIVΔnef. Immune protection takes approximately 15–20 weeks to develop following vaccination [18] and during that time, SIV-specific T cells acquire a more central memory-like phenotype but maintain elevated PD-1 expression [14]. Persistent expression of PD-1 on SIV-specific CD8^+^ T cells requires ongoing low-level viral replication, as PD-1 expression is down regulated on cells specific for an epitope that undergoes escape [14]. These data, in conjunction with evidence of viral evolution following vaccination [1,19–21] and evidence that ongoing replication is required for vaccine efficacy, suggest that continued stimulation from viral epitopes present due to ongoing low-level replication of vaccine virus may induce a unique differentiation or activation state of SIV-specific CD8^+^ T cells. SIVΔnef may also promote a balance or distribution of central memory and effector cells, or a T cell repertoire different than that induced by less protective vaccines. A complete understanding of how SIVΔnef-induced CD8^+^ T cells mediate protection, and the relationship between CD8^+^ T cell differentiation stage and protective immunity remains unclear. Novel experimental methods that can provide additional information to what can be acquired with traditional approaches such as polychromatic flow cytometry may facilitate a more complete characterization of immune protection.

In the past decade, substantial progress has been made in characterizing the differentiation of CD8^+^ effector and memory cell subsets following antigenic stimulation [22]. Genetic approaches have demonstrated the importance of a number of individual transcription factors in regulating differentiation. However, the combinatorial expression of lineage-specific and general transcription factors and their aggregation at *cis*-regulatory elements dictate the expression of any specific gene and ultimately the phenotype of the cell [23–25]. Recognition of the combinatorial nature of transcription factor function has motivated a more holistic approach to understanding transcription factor function and prompted a number of comprehensive systems based descriptions of differential transcription factor usage and networks of transcriptional control in different tissues and cell lineages including the hematopoietic system [23,24,26,27].

To expand on the current capacity for cell phenotypic and functional analyses provided by methods such as polychromatic flow cytometry, we developed a novel approach that exploits the fundamental regulation of cell phenotype and function by combinatorial transcription factor activity. We reasoned that simultaneous expression profiling of multiple transcription factors known to regulate cell differentiation could facilitate discrimination of cell lineage and provide novel information complementary to other methods. To assess the utility of transcription factor expression profiling for the characterization of CD8^+^ T cell differentiation, we measured the expression of a panel of transcription factors known to regulate T cell differentiation in sorted bulk populations of naïve and memory CD8^+^ T cells and in SIV-specific cells induced by SIVΔnef vaccination. Subsequent organization of samples by unsupervised clustering of expression data indicates that transcription factor expression profiling is a sensitive method that can clearly identify cells at different stages of CD8^+^ T cell differentiation. We subsequently applied this method to further characterize the differentiation of CD8^+^ T cells induced by SIVΔnef, and to characterize the phenotype of CD8^+^ T cells temporally associated with either protective, or non-protective immune responses. Our data demonstrate the utility of transcription factor expression profiling to characterize the differentiation of CD8^+^ T cells following SIVΔnef vaccination, and indicate that SIV-specific CD8^+^ T cells appear to be transcriptionally intermediate to, yet clearly distinct from, purified effector memory and central memory T cells isolated from vaccine-naïve animals. Taken together, our results support the conclusion that ongoing activation of CD8^+^ T cells by replicating vaccine virus may induce populations of CD8^+^ T cells possessing phenotypic characteristics distinct from, but with similarities to, classically defined effector memory and central memory cells.

## Results

### Transcription factors selected for expression profiling

To examine whether expression profiling of multiple transcription factors would facilitate discrimination of different CD8^+^ T cell differentiation states, we initially selected a panel of 18 transcription factors to analyze (Table 1) based on published data indicating their involvement in the regulation of CD8^+^ T cell differentiation or function. A subset of these transcription factors, such as T-bet, Eomes, Blimp-1 and Id2 are well-characterized primary regulators of CD8^+^ memory or effector cell differentiation [28–36]. Another set of transcription factors, including BATF, Runx3 and BCL11b, regulate expression of the transcription factors noted above that serve as regulators of T cell function [37–43]. A third group, the Wnt signaling pathway effector transcription factors Lef-1 and Tcf-7 (also known as Tcf-1), are positive regulators of quiescence [44,45]. A fourth set of transcription factors, Rorα, Rorγt and GATA3, regulate differentiation of additional T cell lineages and CD8^+^ T cell activation and effector function [46–49].

**Table 1:** Transcription factors analyzed in bulk and SIV-specific CD8^+^ T cells.

Gene Symbol	Transcription Factor	Function
*PRDM1*	Blimp-1	Represses IL-2 expression, Bcl-6 antagonist, promotes effector differentiation [32,33]
*TBX21*	T-bet	Promotes CD8 effector differentiation [28,29]
*EOMES*	Eomesodermin	Required for early CD8 effector function and subsequent memory CD8 differentiation [30,31]
*BCL6*	Bcl-6	Induces central memory differentiation, functions reciprocally to Blimp-1 [55,61]
*BCOR*	BCoR	Bcl-6 corepressor [54]
*TCF7*	Tcf-7 (Tcf-1)	Wnt effector, promotes quiescence, downregulated with antigen stimulation, promotes Eomes expression [44,45]
*LEF1*	Lef-1	Wnt effector, promotes quiescence, downregulated with antigen stimulation [44]
*RORC*	Rorγt	Represses IFNγ expression, functions as transcriptional repressor in naïve or memory CD8 T cells [46,49]
*AHR*	AHR	Regulates lymphoid tissue inducing function of innate lymphoid cells [84,85]
*RORA*	Rorα	Promotes effector responses in activated CD8 cells [46]
*BATF*	BATF	Upregulated by PD-1 signaling, promotes Rorγt and T-bet expression, regulates effector differentiation; upregulated in exhausted CD8 cells [37–39]
*PBX3*	PBX3	Mediates locus accessibility, upregulated in exhausted CD8 cells [86,87]
*BCL11B*	BCL11b	Regulates Runx3 and FoxP3, promotes cytolytic effector function [41–43]
*RUNX3*	Runx3	Promotes expression of T-bet and Eomes, IFNγ, perforin, granzyme B [40]
*GATA3*	GATA3	Required for sustained TCR-mediated signaling and CTL activity [48]
*ID2*	Id2	Promotes CD8 effector memory differentiation [34–36]
*IRF4*	IRF4	Induced by antigen receptor signaling, represses Eomes, promotes Blimp-1 expression; cooperatively regulates effector differentiation with BATF [39,56,88,89]
*NFIL3*	NFIL3	Activates IL-3 promoter, essential for intraepithelial lymphocyte development [90,91]

### Transcription factors are differentially expressed in sorted CD8^+^ T cell subsets

**Fig 1:**
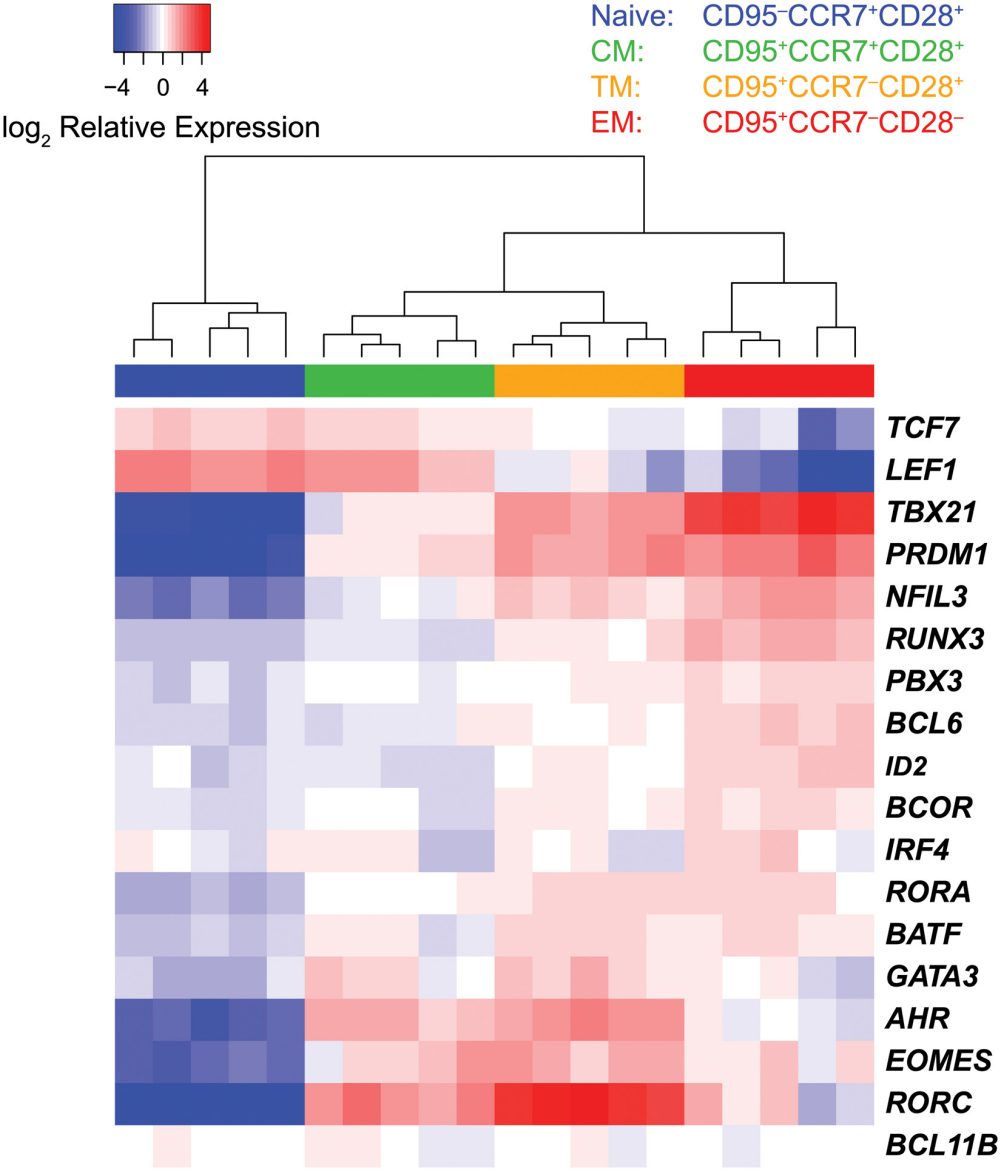
Segregation of sorted CD8^+^ T cell subsets by unsupervised hierarchical clustering. Heat map expression values were transcript mean centered and represent expression relative to endogenous controls. Hierarchical clustering was performed using Euclidean distance and complete linkage methods.

To determine if transcription factor expression profiling can be used to identify distinct stages of CD8^+^ T cell differentiation, we initially sorted highly purified populations of naïve, central memory, transitional memory, and effector memory CD8^+^ T cells from five healthy unvaccinated uninfected rhesus macaques based on differential surface expression of CD28, CD95 and CCR7 (S1 Fig., Fig. 1). We then measured the expression of the transcription factors included in our panel by multi-target qPCR, and used agglomerative unsupervised hierarchical clustering of the expression data to organize the samples. The sample dendrogram (Fig. 1) demonstrates clear segregation of samples by cell differentiation stage, with the three memory CD8^+^ T cell subsets segregating from naïve cells.

**Fig 2:**
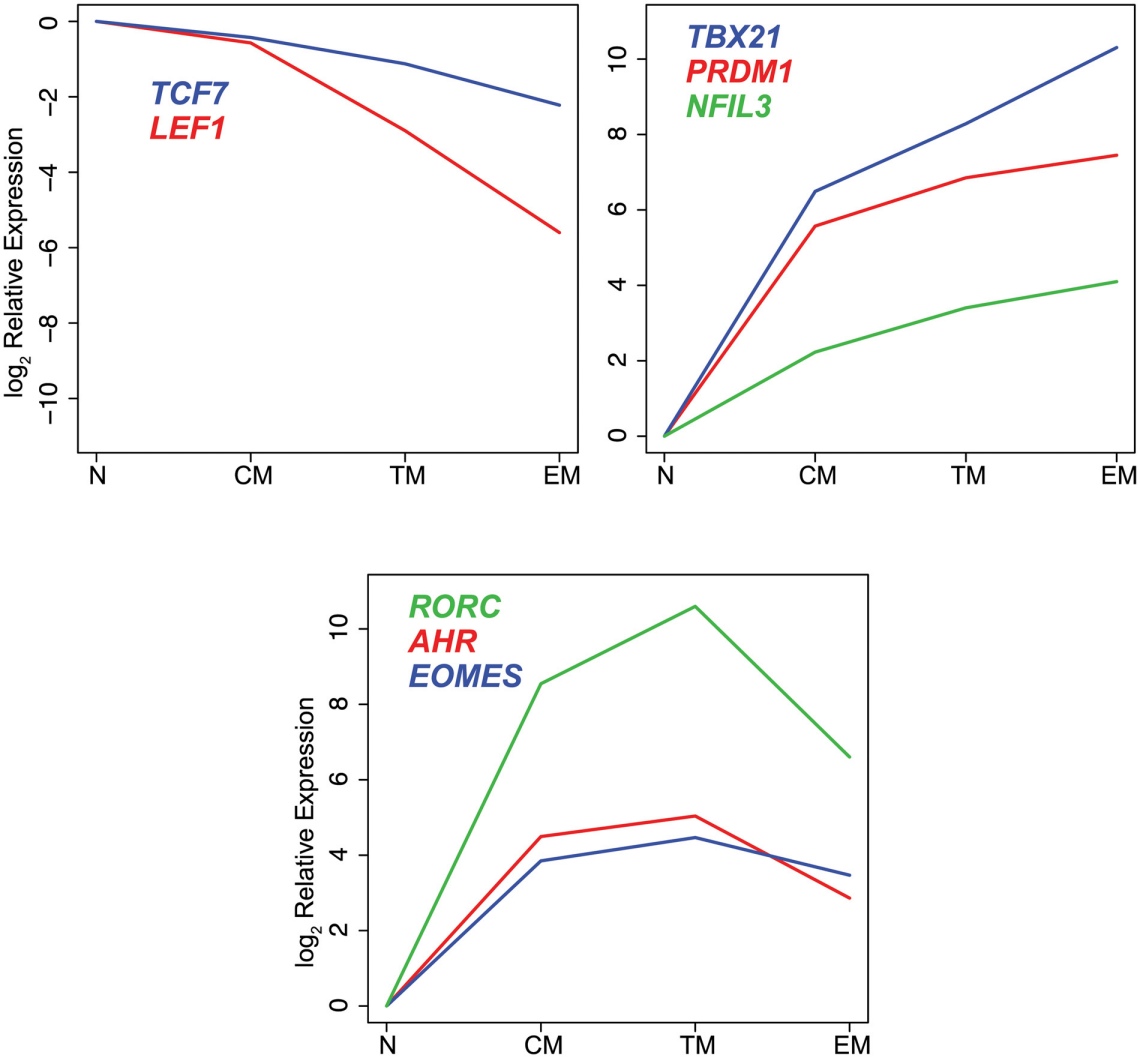
Differential expression of transcription factors in CD8^+^ naïve and memory T cell subsets. log_2_ mean expression values were normalized to naïve cell samples.

Distinct sets of transcription factors displayed unique expression profiles among cell subsets (Fig. 2). The Wnt pathway effectors *LEF1* and *TCF* were expressed at the highest levels in naïve and central memory cells, and lower levels in transitional and effector memory cells. The transcription factors *TBX21*, *PRDM1* and *NFIL3*, were expressed at the highest levels in effector memory cells. In contrast, *EOMES*, *AHR* and *RORC*, were expressed at the highest level in transitional memory cells.

All of the transcription factors except *IRF4* and *BCL11B* were expressed differentially among the CD8^+^ T cell subsets (p≤0.001). The differences in expression levels varied widely among transcription factors with some transcription factors demonstrating up to 1000-fold differences in mean expression level between sorted cell populations.

Unsupervised clustering of samples by differentiation stage demonstrates that expression profiling of transcription factors is a sensitive method that can be used to clearly resolve distinct stages of memory CD8^+^ T cell differentiation.

### SIV-specific CD8^+^ T cells isolated at week 5 or week 20 post-vaccination with SIVΔnef have distinct expression profiles

Longitudinal studies suggest that vaccine-induced protection to pathogenic virus challenge matures during the weeks following vaccination [2,11,18,50]. Animals challenged at 15 to 20 weeks following vaccination are better protected than animals challenged at five weeks following vaccination. As transcription factor expression profiling was able to differentiate between sorted naïve and memory T cell subsets, we sought to use this approach to identify differences in transcription factor usage in SIV-specific CD8^+^ T cells isolated at time points following SIVΔnef vaccination associated with either lesser or greater protection, and to further characterize the phenotype of these cells by comparing their transcription factor expression profiles with the profiles of sorted naïve and memory CD8^+^ T cell subsets. We analyzed CD8^+^ T cells specific for either of two Mamu-A*01-restricted immunodominant SIV epitopes differing in their propensity for immune escape. The Gag CM9 epitope is typically conserved over time [51], whereas the Tat SL8 epitope mutates rapidly following infection in response to immune pressure, beginning to accumulate sequence heterogeneity at two weeks post infection [52,53]. We hypothesized that the distinct escape kinetics and resulting sensitivities to ongoing antigenic stimulation would induce differences in differentiation stage resolvable by transcription factor expression profiling. We sorted Gag CM9- and Tat SL8- specific CD8^+^ T cells obtained from four rhesus macaques at either 5 weeks or 20 weeks following SIVΔnef vaccination, and measured the expression levels of the transcription factors in our target panel by multi-target qPCR.

**Fig 3:**
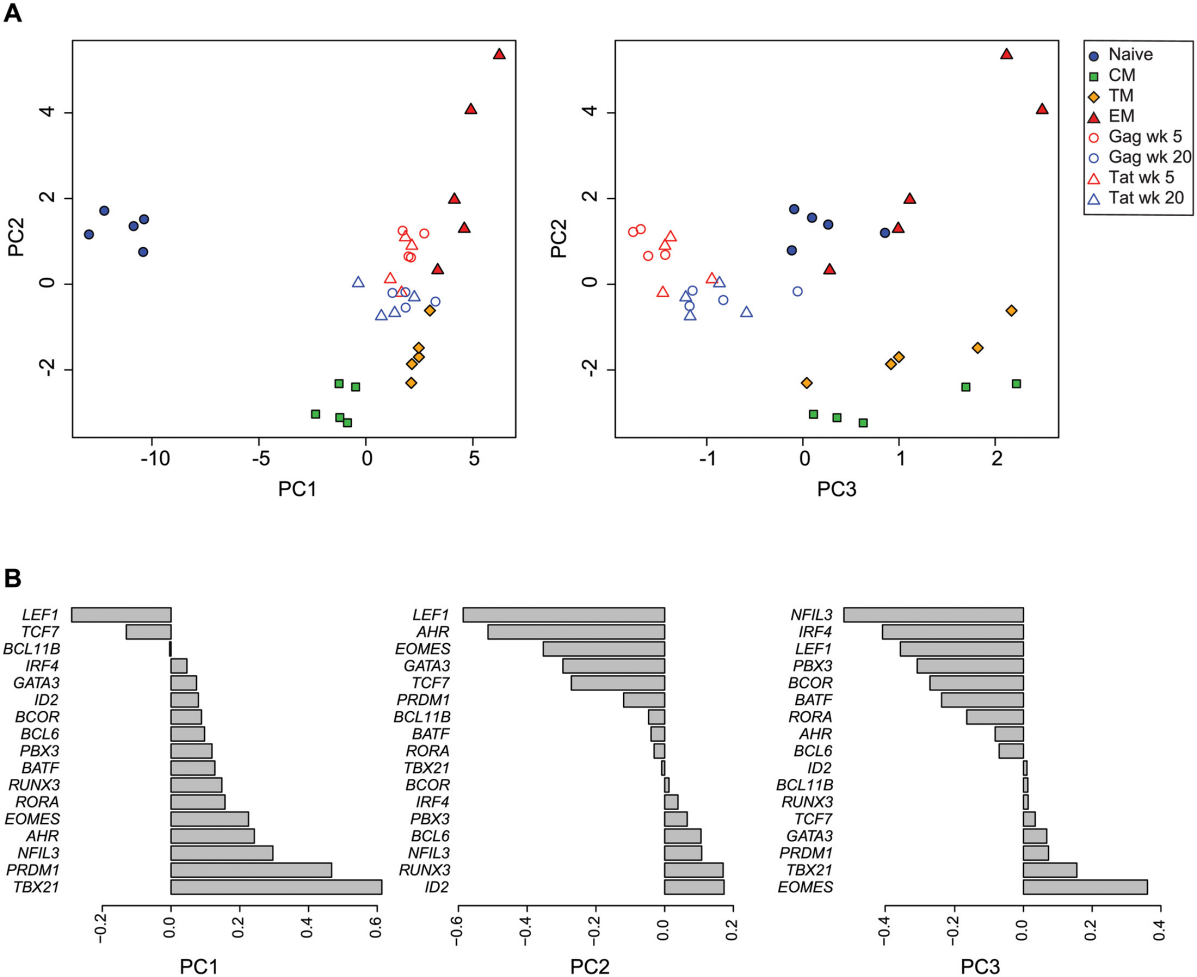
Principal component analysis of transcription factor expression profiles from SIV-specific MHC tetramer-sorted CD8^+^ T cells and sorted CD8^+^ T cell subsets. (**A**) Plot of principal components 1 vs. 2, and 2 vs. 3 for each of the expression profiles assessed in sorted naïve and memory CD8^+^ T cell subsets isolated from healthy control animals (n = 5), and SIV-specific MHC tetramer-sorted CD8^+^ T cells isolated from animals (n = 4) at week 5 or week 20 following SIVΔnef vaccination. Principal components 1, 2 and 3 explain 92% of cumulative total variance. (**B**) PCA loading factors for each transcription factor.

To integrate the expression profiles of the SIV-specific cells with the sorted CD8^+^ subsets, we applied principal component analysis (PCA) to the combined data sets. Plotting principal components 1 vs, 2, and principal components 2 vs. 3, (PC1, PC2, PC3; Fig. 3A, S1 Video) segregated the data into distinct clusters. The data points representing the sorted CD8^+^ T cells occupy the periphery of the PC1 vs. PC2 plot, and segregate into separate clusters based upon cell differentiation stage. The naïve cells segregate from the memory cells along the PC1 axis, whereas the memory cells segregate along the PC2 axis, with the transitional memory cells positioned intermediately between the central and effector cells. The PC1 and PC2 loading factors (Fig. 3B) indicate that in this analysis, differential expression of *LEF1*, *TCF7*, *PRDM1* and *TBX21* strongly influence segregation of naïve from memory cells, whereas differential expression of *ID2*, *RUNX3*, *AHR* and *LEF1* strongly influence segregation of memory cell subsets. The SIV-specific CD8^+^ T cells cluster with the sorted memory cells on the PC1 axis, and are positioned intermediately between central memory and effector memory cells on the PC2 axis. This intermediate position on the PC2 axis in part reflects the combined expression profiles of different memory subsets present in the SIV-specific cell samples. However, the SIV-specific samples significantly segregate from any sorted memory subset, particularly on the PC3 axis (p<0.001), indicating that the transcription factor expression profiles of the SIV-specific cells are distinct from the sorted subsets and are not solely comprised of proportions of memory subsets. The PC3 loading factors (Fig. 3B) indicate that in this analysis, the differential expression of *NFIL3*, *IRF4*, *LEF1* and *EOMES* influence segregation of SIV-specific cells from the sorted naïve and memory subsets. The SIV-specific cells form two clusters, generally organized by week post-infection. The week 5 and week 20 post-vaccination samples occupy significantly different positions in PCA space (p<0.01). The week 5 post-vaccination cells, temporally associated with less protection to challenge, have greater PC2 values, indicating a more effector-like profile, whereas the week 20 post-vaccination cells have lesser PC2 values indicating a more central-memory like profile. The samples also significantly segregate based on epitope specificity (p<0.01). The Gag CM9-specific cells have overall higher PC2 values indicating a more effector-like phenotype, whereas the Tat SL8-specific cells have lower PC2 values indicating a more central memory-like phenotype. The changes in expression profiles from week 5 to week 20 are consistent with SIV-specific CD8^+^ T cells becoming overall more central memory-like and less effector-like over time following vaccination. Similarly, the differences seen between Gag CM9- and Tat SL8-specific cells are consistent with the kinetics of epitope escape and likely reflect the loss of antigen stimulation of the Tat-specific cells versus the ongoing stimulation of the Gag-specific cells.

To further validate the approach of using transcription factor expression profiling to characterize cell differentiation, we compared this method to conventional flow-cytometric assessment of CD8^+^ T cell memory subsets present in SIV-specific populations at different time points following vaccination. In agreement with our expression profiling methods, flow cytometric methods based on differential expression of CCR7 and CD28 showed an overall increase in CM cells and a decrease in EM cells from week 5 to week 20 post-vaccination. Furthermore, Tat SL8-specific cells had greater frequencies of CM cells and lesser frequencies of EM cells than Gag CM9-specific cells (S2A Fig.). Additionally, the ratio of EM to CM cells found in a sample of SIV-specific cells positively correlates with the PC2 value of the combined PCA analysis (S2B Fig.).

To provide additional context for interpreting SIV-specific CD8^+^ T cell expression profiles, and to further examine the effect of ongoing viral replication on transcription factor expression profiles, we used PCA to compare vaccine-specific and sorted naïve and memory subsets to SIV-specific CD8^+^ T cells collected at week 20 following infection with pathogenic wild-type SIV.

**Fig 4:**
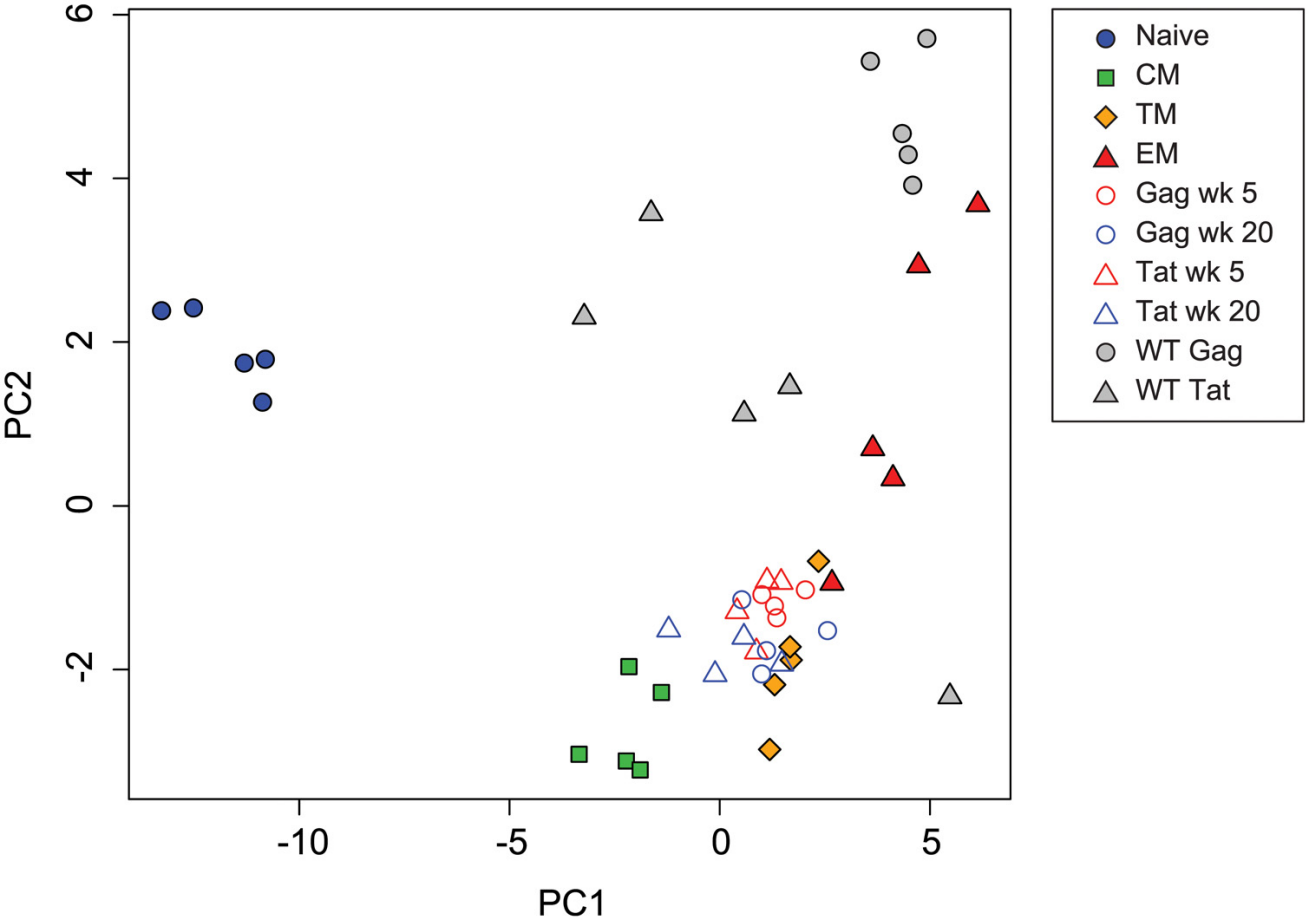
Principal component analysis of transcription factor expression profiles from SIV-specific MHC tetramer-sorted CD8^+^ T cells from animals vaccinated with SIVΔnef, animals infected with wild-type SIV, and sorted CD8^+^ T cell subsets. Plot of principal components 1 and 2 for each of the expression profiles assessed from sorted naïve and memory CD8^+^ T cell subsets, SIV-specific MHC tetramer-sorted CD8^+^ T cells isolated from SIVΔnef-vaccinated animals, and MHC tetramer-sorted CD8^+^ T cells isolated from animals (n = 5) at 20 weeks following wild-type SIV infection. Principal components 1 and 2 explain 77 percent of cumulative total variance.

A plot of principal components 1 and 2 (Fig. 4) positioned Gag CM9-specific cells obtained from wild-type SIV-infected animals near the sorted effector memory cells but with higher PC2 values. Tat SL8-specific cells from wild-type SIV-infected animals were more heterogeneous but generally occupied positions more positive along the PC2 axis than SIVΔnef-induced cells, with PC2 values similar to sorted effector memory cells. These results are consistent with the idea that ongoing activation by replicating virus may induce a more effector-like phenotype, and that epitope escape facilitates a more central memory-like phenotype.

**Fig 5:**
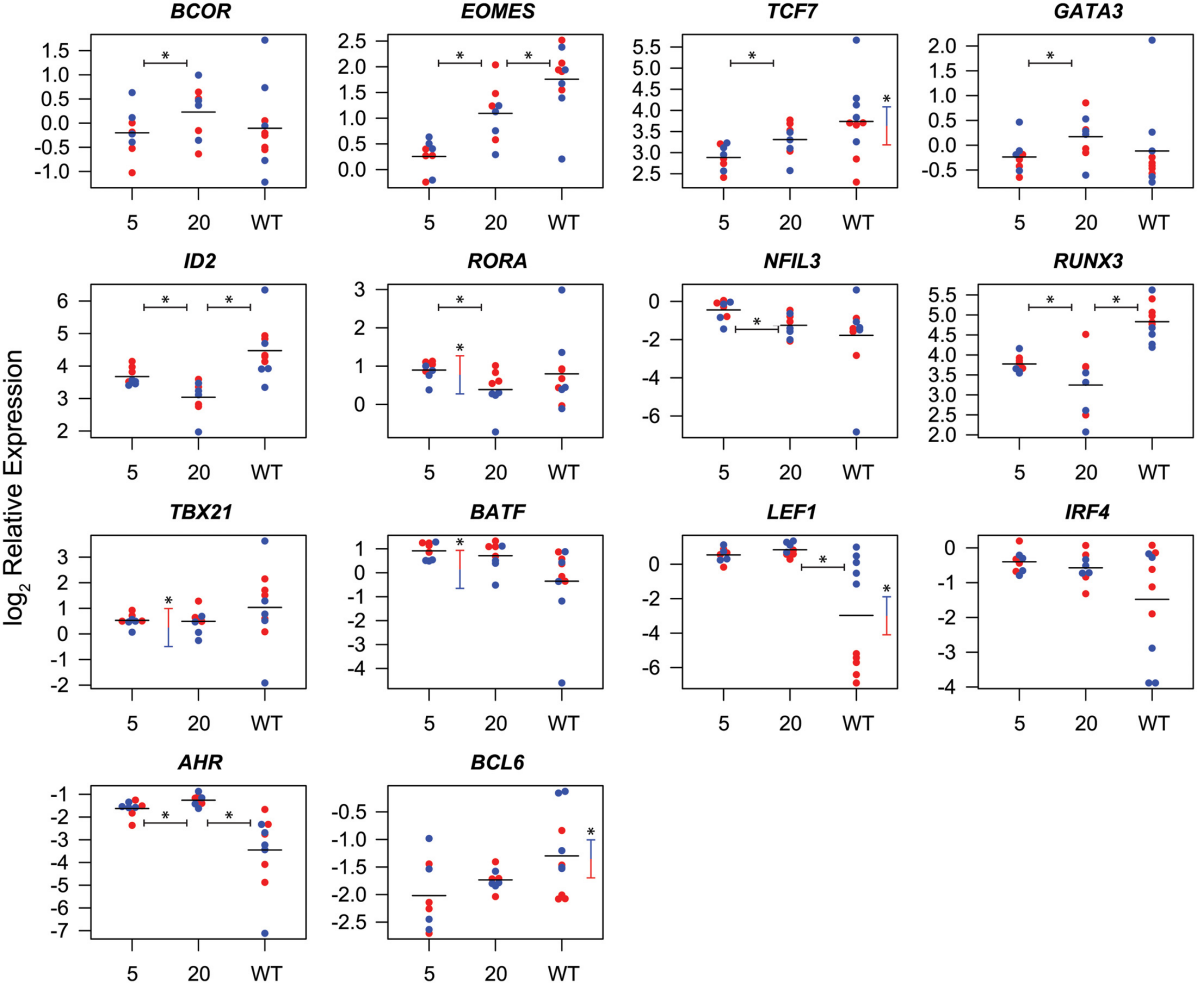
Differential expression of transcription factors in CD8^+^ T cells isolated at week 5 and week 20 post-vaccination with SIVΔnef and at week 20 post-infection with wild-type SIV. Symbols indicate log_2_ expression relative to endogenous controls in cells from individual animals. Red symbols indicate Gag CM9-specific cells, blue symbols indicate Tat SL8-specific cells. Sample means are indicated by horizontal bars. Statistically significant (p≤0.05) differences in transcription factor expression between cells from week 5 and week 20 post-SIVΔnef vaccination, or between cells from week 20 post-SIVΔnef vaccination and week 20 post-wild-type SIV infection are indicated by horizontal bars with asterisks. Statistically significant differences between Gag CM9 and Tat SL8-specific cells are indicated by vertical bars with asterisks.

A number of individual transcription factors were significantly differentially expressed between week 5 post-vaccination and week 20 post-vaccination (Fig. 5). For example *BCOR*, *EOMES* and *TCF7* were expressed at significantly higher levels at week 20 post-vaccination. Higher expression of these transcription factors is consistent with a more quiescent and central memory-like phenotype [44,45,54]. Conversely, *ID2*, *RORA*, *NFIL3* and *RUNX3* were expressed at significantly lower levels at week 20 post-vaccination. Lower expression of *ID2* and *RUNX3* is also consistent with a more central memory-like phenotype [35,40]. Elevated *TBX21* expression, which is associated with effector differentiation, however, was maintained at week 20, consistent with continued effector function [28,29].

A number of transcription factors were also significantly differentially expressed between Gag CM9- and Tat SL8- specific cells. Gag-specific CD8^+^ T cells expressed significantly higher levels of *BATF*, *TBX21* and *RORA*. These differential expression profiles are consistent with Gag CM9-specific cells maintaining a more effector-like phenotype than Tat SL8-specific cells [28,37,46]. Interestingly, *EOMES* was expressed at significantly lower levels in Gag CM9-specific cells than in Tat SL8-specific cells at week 5 post-vaccination, and a trend towards higher expression was observed in Gag CM9-specific cells than in Tat SL8-specific cells at week 20 post-vaccination. Conversely, *ID2* is expressed at significantly higher levels at week 5 in Gag-specific cells and more similar to Tat-specific cells at week 20. These trends are consistent with Tat-specific cells being more central memory-like even at the earlier time point [31,35].

Many of the transcription factors were differentially expressed between SIV-specific CD8^+^ T cells in wild-type SIV- and SIVΔnef-infected animals at week 20 post-vaccination, likely reflecting the higher viral loads and attendant greater stimulation of SIV-specific cells in animals infected with wild-type SIV. *EOMES*, *ID2* and *RUNX3* were expressed at significantly higher levels in cells from wild-type SIV-infected animals (p≤ 0.05). In contrast, *AHR* and *LEF1* were expressed at significantly lower levels in wild-type SIV-specific cells. A number of transcription factors were differentially expressed between Gag- and Tat-specific cells in wild-type SIV-infected animals. In particular, the Wnt pathway effectors *TCF7* and *LEF1*, as well as *BCL6*, had significantly higher expression in Tat-specific cells. In contrast, *EOMES*, *RUNX3* and *IRF4* demonstrated trends towards higher expression in Gag-specific cells. These differences are consistent with the different kinetics of epitope escape, and consistent with loss of antigen stimulation mediating cell differentiation towards a less activated or more central memory phenotype [11,12,44,55,56].

Overall, the differences we observed in transcription factor expression profiles suggest that the process of CD8^+^ T cell differentiation following SIVΔnef vaccination involves the coordinate regulation of multiple transcription factors. At later time points following vaccination, SIV-specific cells express higher levels of transcription factors associated with memory differentiation, such as *EOMES* and *TCF7*, down-regulate transcription factors associated with effector responses such as *ID2* and *RUNX3*, yet maintain elevated levels of *TBX21*.

**Fig 6:**
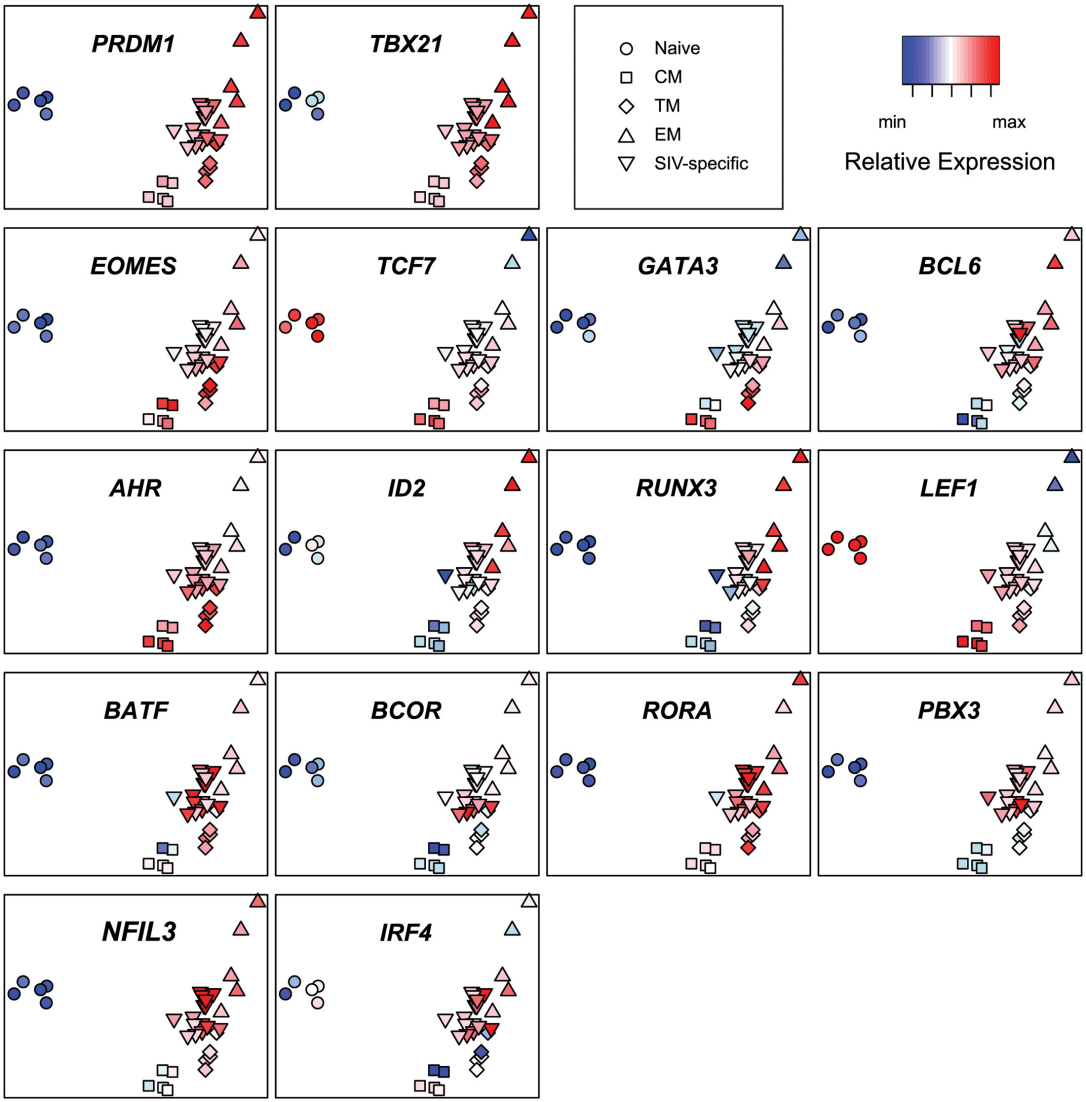
Principal component analysis heat maps of transcription factor expression profiles from SIV-specific MHC tetramer-sorted CD8^+^ T cells from animals vaccinated with SIVΔnef, and sorted naïve and memory CD8^+^ T cells. Heat map log_2_ relative expression values were range normalized for each transcription factor.

To facilitate comparison of expression levels of individual transcription factors in the different populations of T cells, we also generated a PCA heatmap (Fig. 6) of each transcription factor by overlaying expression values as heatmap colors on a plot of principal components 1 and 2.

The PCA heatmaps demonstrate that expression of *PRDM1* and *TBX21* in SIV-specific cells is similar to central memory or transitional memory cells. In contrast, expression of *EOMES*, *TCF7*, *GATA3* and *BCL6* in SIV-specific cells is more similar to effector memory cells. Expression of *AHR*, *ID2*, *RUNX3* and *LEF1* in SIV-specific cells is intermediate between central memory and effector memory cells. Interestingly, expression of the transcription factors *BATF*, *BCOR*, *RORA*, *NFIL3*, *IRF4* and *PBX3* is greater in SIV-specific cells than any sorted subset. The higher expression levels of these transcription factors in SIV-specific cells provides additional evidence that the SIV-specific cells are transcriptionally distinct from any purified memory subset, or proportion of subsets. Although statistically significant for only *PBX3* (p<0.05) these trends of increased expression may, in part, reflect ongoing stimulation from replicating virus.

## Discussion

Memory T cells display substantial heterogeneity in phenotype, function and anatomic distribution [57]. The characterization of memory cell differentiation and the definition of phenotypic memory cell subpopulations has traditionally employed flow cytometric analyses of a subset of cell surface proteins, which regulate cell activation, survival and tissue-homing [58–60]. Over the past decade, the characterization of molecular mechanisms regulating memory differentiation has also identified key transcription factors that modulate the gene expression profiles in differentiating cells and cell subpopulations [22]. As transcription factors fundamentally regulate cell phenotype and function, global analyses of differential transcription factor usage accompanying cell differentiation can conceptually both identify novel subpopulations of cells not resolvable by standard flow cytometric techniques and provide novel insights into the process of cell differentiation and the function of cell subpopulations.

We validated this approach by initially characterizing the transcription factor expression profiles of CD8^+^ T cells isolated at different stages of CD8^+^ T cell differentiation, and we subsequently used these data to characterize CD8^+^ T cells induced by SIVΔnef vaccination and wild-type SIV infection. Clustering analyses of transcription factor expression data verified that CD8^+^ T cells purified by traditional flow cytometric gating strategies display distinct transcription factor expression profiles. This strict segregation by differentiation state, of cells from all animals is striking, given that rhesus macaques are outbred animals and would be expected to display substantial transcriptional heterogeneity between individuals. The three memory subsets had transcription factor expression profiles more similar to each other than to naive cells, and the transitional memory cells appeared more similar to central memory cells than to effector memory cells. The expression profiles across subsets corroborates, for many of the transcription factors, patterns of expression that have been reported for single transcription factors or small subsets of transcription factors, although comprehensive analyses of expression of multiple transcription factors have not previously been undertaken. *TBX21*, *PRDM1*, *RUNX3* and *ID2*, for example, were expressed at higher levels in sorted effector memory cells, whereas the Wnt pathway effectors *LEF1* and *TCF7* were expressed at higher levels in the more quiescent naive and central memory cells. The high ratio of *TBX21* to *EOMES* observed in effector memory cells, and similarly, the high ratio of *PRDM1* to *BCL6* observed in transitional memory and effector memory cells is consistent with the promotion of an effector phenotype [61]. Given the caveat that differences in transcription do not always directly correlate with differences in protein expression or function, overall, these results validate the approach of using transcription factor expression profiling to define the memory differentiation state of CD8^+^ T cells.

We subsequently used transcription factor expression profiling to characterize changes in SIV-specific CD8^+^ T cells over time as immune protection to challenge matures following vaccination. Half of the transcription factors assessed had significantly different (p≤0.05) expression levels at week 20 than at week 5 post-vaccination, and segregation by PCA suggested that SIV-specific cells had substantially different expression profiles at the two time points. SIV-specific CD8^+^ T cells in SIVΔnef-vaccinated animals at week 20 displayed a transcriptional signature characteristic of (but not identical to) central memory cells, as manifested by elevated levels of *TCF7* and *EOMES* and decreased expression of *ID2* and *RUNX3*. Importantly, because transcription was assessed in bulk populations of cells, differences between levels of expression, and location in PCA space reflect both differences between expression levels on a per-cell basis, and differences in proportions of subsets present in the sample. Thus, the more central memory-like expression profile displayed at week 20 post-vaccination likely reflects the greater proportion of central memory-like cells present at week 20 post-vaccination, in agreement with our conventional flow cytometric analyses, but may also reflect a more central-memory like profile in SIV-specific cells overall.

A number of transcription factors were also differentially expressed between cells with different epitope specificities and immune escape kinetics. For example, *TBX21* and *BATF* were expressed at higher levels in Gag CM9-specific cells than in Tat SL8-specific cells. Since BATF is upregulated downstream of PD-1 signaling [38], this observation is consistent with the higher levels of PD-1 expressed on Gag CM9-specific cells than in Tat SL8-specific cells [14]. The role of PD-1 in promoting an exhausted T cell phenotype in the setting of chronic viral infection has been widely described [62,63]. However, the expression of PD-1 is not an indication in itself of an exhausted phenotype and more accurately reflects cell activation [13,14,64]. Overall, the Tat SL8-specific cells appeared less activated and more memory-like than the Gag CM9-specific cells. Although differences in expression profiles between Gag CM9- and Tat SL8-specific cells are likely to be influenced by a variety of variables, the differences we observed are consistent with the loss of antigenic stimulation due to the evolution of escape in the Tat SL8 epitope, and on-going stimulation of Gag CM9-specific cells. These results are also consistent with prior work demonstrating a decline in the frequency of activated Tat SL8-specific cells, but not Gag CM9-specific cells in genital tissue from 5 to 20 weeks post-vaccination with SIVΔnef [12].

SIV-specific cells from wild-type SIV-infected animals displayed a pattern of expression of transcription factors more characteristic of effector memory cells than SIV-specific cells from SIVΔnef-vaccinated animals. Gag CM9-specific cells more closely resembled effector memory cells in expression of transcription factors than Tat SL8-specific cells, as predominantly reflected by their differential expression of *LEF1* and *TCF7*. Further, *LEF1* was expressed at overall lower levels in cells from wild-type-infected animals than SIVΔnef-vaccinated animals, mainly due to very low expression in the Gag CM9-specific cells. This difference likely reflects the much higher levels of antigen stimulation in wild-type SIV-infected animals. *BATF* was also expressed at lower levels in cells from wild-type-infected animals than in cells from SIVΔnef-vaccinated animals. Since BATF has recently been shown not only to promote effector differentiation, but also to restrain the expression of the effector molecules IFNγ and granzyme B [39], the reduced expression observed in wild-type-infected animals may reflect reduced inhibition of effector molecule expression in the presence of high viral loads. Overall, these results are consistent with previous studies suggesting virus-specific cells continue to be activated by replicating virus, and that CTL escape is associated with reduced cell activation and central memory differentiation [3,12–14].

Studies in macaques using the live-attenuated SHIV89.6 vaccine suggest that protection against vaginal challenge is associated with the presence of SIV-specific CD8^+^ T cells in the female reproductive tract that possess both cytolytic function and some proliferative capacity [65,66]. Earlier studies showed that protection was associated with a higher ratio of central memory to effector memory CD8^+^ T cells in blood and lymph nodes [67]. CD8^+^ T cells from protected animals also showed higher pre-challenge measures of survival and lower apoptotic potential. In contrast to the effector memory cells found in the female reproductive tract, IL-2 secreting SIV-specific CD8^+^ T cells were found in lymph nodes. Overall this suggests a model whereby in protected animals, SHIV89.6 induces central memory CD8^+^ T cells that continually supply effector cells to the genital mucosa in response to persistent antigenic stimulation by replicating virus. That unprotected animals have a higher ratio of effector memory to central memory cells in blood and lymph nodes suggests that lack of protection may be associated with heightened systemic T cell activation and resultant apoptosis and exhaustion.

In the setting of spontaneously controlled HIV infection, overwhelming evidence suggests CD8^+^ T cell activity is critical for viral suppression. However there has been substantial heterogeneity reported in ex vivo measures of CD8^+^ T cell function in controllers. Studies have variously shown associations between control of viremia and CD8^+^ T cell polyfunctionality, HIV-specific CD8^+^ T cell frequency, virus suppressive capacity, or proliferative capacity [68–72]. A more recent study [73] examined individuals who control viremia to very low levels in the absence of ex vivo CD8^+^ T cell responses (weak responders), and found that these subjects maintain an HIV-specific population of central memory CD8^+^ T cells capable of suppressing HIV ex vivo. An additional study [74] showed that spontaneous protection from HIV in controllers correlates with CD8^+^ T cell memory-type responses, prolonged cytokine secretion, and cell proliferation. In the setting of elite control and very low viremia, HIV-specific T cells may receive less antigenic stimulation facilitating differentiation towards a more central memory phenotype.

Our data demonstrate the utility of using transcription factor expression profiling to characterize the differentiation of CD8^+^ T cells following vaccination with SIVΔnef. Using this approach we demonstrate that SIV-specific cells isolated from vaccinated animals at time points associated with greater immune protection display a distinct pattern of expression of transcription factors that represent the presence of different proportions of CD8^+^ T cell memory subsets, or different levels of activation or differentiation states. The higher expression levels of a number of transcription factors in SIV-specific cells than in any purified memory subset suggests that continued activation of subsets of virus-specific cells by low-level replicating virus induces transcriptionally distinct populations of CD8^+^ T cells which may have characteristics of both central memory and effector memory cells.

## Materials and Methods

### Ethics statement

The 14 female Indian-derived rhesus macaque monkeys (*Macaca mulatta*) described in this study were housed at the New England Primate Research Center (NEPRC) in accordance with the regulations of the American Association of Accreditation of Laboratory Animal Care and the standards of the Association for Assessment and Accreditation of Laboratory Animal Care International. All protocols and procedures were approved by the relevant Institutional Animal Care and Use Committee, which was the Harvard Medical Area (HMA) Standing Committee on Animals at Harvard Medical School. All animals were housed indoors in an SOP-driven, AAALAC-accredited facility. Husbandry and care met the guidance of the Animal Welfare Regulations, OLAW reporting and the standards set forth in The Guide for the Care and Use of Laboratory Animals. All research animals were enrolled in the NEPRC behavioral management program, including an IACUC-approved plan for Environmental Enrichment for research primates. This program included regular behavioral assessments, and provision of species appropriate manipulanda, and foraging opportunities. This protocol had an IACUC-approved exemption from social housing based on scientific justification. Primary enclosures consisted of stainless steel primate caging provided by a commercial vendor. Animal body weights and cage dimensions were regularly monitored. Overall dimensions of primary enclosures (floor area and height) met the specifications of The Guide for the Care and Use of Laboratory Animals, and the Animal Welfare Regulations (AWR’s). Further, all primary enclosures were sanitized every 14 days at a minimum, in compliance with AWRs. Secondary enclosures (room level) met specifications of The Guide with respect to temperature, humidity, lighting and noise level. The animals were provided ad lib access to municipal source water, offered commercial monkey chow twice daily, and offered fresh produce a minimum of three times weekly. Light cycle was controlled at 12/12 hours daily. The animals were subject to twice daily documented observations by trained animal care and veterinary staff, and enrolled in the facility’s environmental enrichment, and preventative health care programs. Euthanasia took place at defined experimental endpoints using protocols consistent with the American Veterinary Medical Association (AVMA) guidelines. Animals were first sedated with intramuscular ketamine hydrochloride (20 mg/kg) followed by sodium pentobarbital (≥100 mg/kg) intravenously to achieve euthanasia.

### Isolation of lymphocytes

Peripheral blood samples were collected from unvaccinated healthy rhesus macaques (n = 5) for purification of naïve and memory CD8^+^ T cell subsets, or Mamu-A*01^+^ SIVΔnef-vaccinated animals (n = 4) at week 5 and week 20 post-vaccination, or Mamu-A*01^*+*^ wild-type SIV-infected animals (n = 5) at week 20 post-infection, for purification of SIV-specific cells. Blood was collected in EDTA vacutainer tubes (Becton Dickinson Vacutainer systems, Franklin Lakes, NJ), and peripheral blood mononuclear cells (PBMC) were separated by density gradient centrifugation (Lymphocyte Separation Medium; MP Biomedicals Inc., Solon, OH) at 1500 rpm for 45 minutes. PBMC from vaccinated or infected animals were cryopreserved, and subsequently thawed prior to cell sorting. PBMC from healthy uninfected animals were used immediately after separation.

### Plasma viral load quantification

Total RNA copy number equivalents were determined in EDTA-treated plasma using a standardized quantitative real-time RT-PCR assay based on amplification of conserved *gag* sequences as described previously [75]. In wild-type SIV-infected animals, viral loads were between 22,000 and 1,900,000 copy equivalents/ml plasma at 20 weeks following infection.

### Cell sorting

Naïve (CD95^−^CCR7^+^ CD28^+^), central memory (CD95^+^ CCR7^+^ CD28^+^), transitional memory (CD95^+^ CCR7^−^CD28^+^) and effector memory (CD95^+^ CCR7^−^ CD28^−^) CD8^+^ T cell subsets were sorted from PBMC from uninfected animals. SIV-specific CD8^+^ T cells were sorted from previously cryopreserved PBMC from SIVmac239Δnef-vaccinated or SIVmac239-infected animals. SIV-specific CD8^+^ T cells from Mamu-A*01^+^ or Mamu-A*02^+^ animals were identified using APC- or PE- conjugated Mamu-A*01 or A*02 MHC class I tetramers or pentamers (Proimmune) complexed with the cognate CTL epitope. A*01 Gag_181–189_CM9 [76] and A*01 Tat_28–35_SL8 [77,78] tetramers were kindly provided by Nancy Wilson and David Watkins (Wisconsin National Primate Research Center, Madison, WI). To identify naïve and memory phenotypes, PBMC were stained with CD3 (SP34) FITC or Pacific Blue; CD4 (L200) PerCP-Cy5.5; CD8 (RPA-T8) Alexa 700; CD28 (28.2) ECD (Beckman Coulter), CD95 (DX2) APC, CCR7 (150503) PE (R&D Systems). Antibodies were obtained from BD Pharmingen unless specified. PBMCs (1–2 × 10^6^) were initially labeled with LIVE/DEAD viability stain (Life Technologies) and washed; incubated with CCR7 antibody for 15 min at 37C; incubated with tetramers or pentamers for 10 min at RT and washed; then incubated with all other antibodies for 20 min at RT and washed prior to sorting. Cell sorting was performed on a FACS Aria II cell sorter (BD Biosciences). Sorts were > 99% pure for all populations, and cell yields generally ranged between 10^3^ and 10^5^ cells.

### RNA extraction, cDNA synthesis and real time PCR

Total RNA was isolated using RNeasy Plus Micro Kit (74034, Qiagen) and quantified from CD8^+^ T cells that were FACS purified from PBMC. cDNA was synthesized using the High-Capacity Reverse Transcription Kit with RNase Inhibitor (4374966, Life Technologies). The resulting cDNA (1ng equivalent input) per reaction was subjected to 18 cycles of preamplification using the ABI Preamp Master Mix kit and pooled TaqMan assays (S1 Table) (Applied Biosystems, Life Technologies). Preamplified cDNAs were diluted 5-fold with 1×TE and loaded on 96x96 Fluidigm BioMark dynamic arrays (Fluidigm) along with the selected real-time PCR assays. All possible combinations of samples and assays on the BioMark dynamic array chip were mixed using the Fluidigm (IFC) integrated fluidic circuit controller. The Fluidigm Biomark System was used for real time PCR amplification and data collection, using 40 cycles of amplification with real-time monitoring of FAM fluorescence in each well. Initial calculations of cycle thresholds (Ct) were performed using the Fluidigm BioMark software and further analysis was carried out using GenEx software (MultiD Analyses, URL: http://www.multid.se). Offscale-low expression values were set to maximum onscale Ct+1 for each target transcript. Five endogenous control genes were included in each Fluidigm run and the stability of endogenous control genes across all experimental samples was analyzed using the NormFinder algorithm in GenEx. The mean expression of the two most stable endogenous control genes (*PGK1* and *TBP*) was used for normalization. Relative expression (2^-Δct^) values were log_2_ transformed for subsequent analyses (S1 Dataset).

### Cluster analyses

Unsupervised agglomerative hierarchical clustering was performed on transcript mean-centered expression values using the Euclidean distance metric and complete linkage clustering method. Principal component analysis was performed using un-scaled expression values. PCA heatmap expression values were normalized by scaling expression values to the range of each transcript. *RORC* was excluded from PCA analyses because *RORC* was expressed at or below the limit of detection in the majority of SIV-specific cells, and chip-to-chip differences in the values assigned to offscale-low reactions introduced artifacts of apparent differential expression that confounded PCA plot structure. Clustering analyses were performed using R [79], and the functions prcomp {stats}, and hclust {stats}. In addition to base R the following R packages were used: RColorBrewer [80], Plotrix [81], gplots [82], lme4 [83].

### Statistical analyses

Statistical analyses were performed using both Stata software (StataCorp. 2013. *Stata Statistical Software*: *Release 13*. College Station, TX: StataCorp LP) and R [79]. Differences in transcription factor expression between sorted naïve and memory subsets, and differences between sorted subsets and SIV-specific cells were assessed by one-way ANOVA. Differences in principal component plot positions of SIV-specific cells and sorted naïve and memory cell subsets were assessed by unpaired Student’s *t*-test of PC3 values. Differences in principal component plot positions of week 5 and week 20 post-vaccination samples and of Gag CM9- and Tat SL8-specific samples were assessed by mixed effects linear regression modeling of PC1 values of a principal component analysis of SIV-specific cells. Differences between individual transcription factor expression values at week 5 and week 20 post-vaccination or infection, or between Gag CM9- and Tat SL8-specific cells, were assessed using mixed effects linear regression models. Differences between individual transcription factor expression values in cells isolated at 20 weeks post SIVΔnef vaccination and wild-type SIV infection were assessed by unpaired Student’s *t*-test. Differences in frequencies of CD8^+^ T cell memory subsets in Gag CM9- and Tat SL8-specific cells were assessed by unpaired Student’s *t*-test (week 5 vs. week 20) or paired Student’s *t*-test (Gag vs. Tat.)

### Target transcript and Entrez Gene ID

*AHR* 714254

*BATF* 702646

*BCL6* 708736

*BCL11B* 705238

*BCOR* 698644

*PRDM1* 696757

*EOMES* 704711

*ID2* 693394

*RORC* 717052

*RORA* 704014

*PBX3* 711691

*NFIL3* 704757

*IRF4* 722883

*RUNX3* 719447

*TBX21* 694044

*TCF7* 710234

*LEF1* 695776

*GATA3* 713840

## Supporting Information

S1 Table TaqMan assays. The qPCR primer/probe sets (TaqMan assays) used to quantify mRNA levels of the indicated target transcripts.
(PDF)

S1 Dataset Expression data. qPCR expression data (log_2_ 2^-Δct^).
(XLSX)

S1 Fig Representative FACS gating. CD8^+^ naïve and memory T cell subsets (A) and Gag CM9 and Tat SL8 tetramer/pentamer-sorted CD8^+^ T cells (B).
(EPS)

S2 Fig Memory phenotype of SIV-specific CD8^+^ T cells and correlation of phenotype with transcription factor expression profile. **(A)** Frequencies of CM (CCR7^+^ CD28^+^), TM (CCR7^-^ CD28^+^), and EM (CCR7^-^ CD28^-^) populations present in Gag CM9- and Tat SL8-specific CD8^+^ T cells were analyzed by flow cytometry. Statistically significant (p≤0.05) differences in frequencies are indicated by bars with asterisks. **(B)** Pearson’s correlation between the ratio of %EM to %CM cells and the transcription factor expression profile PCA PC2 value for each sample of Gag CM9- and Tat SL8-specific CD8^+^ T cells.
(EPS)

S1 Video Principal Component Analysis of PC1, PC2 and PC3. Naïve (dark blue), EM (red), TM (orange), CM (green), week 5 p.v. (pink) and week 20 p.v. (light blue) SIV-specific cell samples are presented.
(MP4)
