# Correction: Characterization of CD8+ T Cell Differentiation following SIVΔnef Vaccination by Transcription Factor Expression Profiling

**DOI:** 10.1371/journal.ppat.1004968

**Published:** 2015-06-11

**Authors:** The PLOS Pathogens Staff

## Notice of Republication

This article was republished on May 19, 2015, to correct the figures that were published in low resolution. This error was introduced during the typesetting process, and the publisher apologizes for the errors. Please download this article again to view the correct version.
