# Supplementary material for: Characterization of CD8+ T Cell Differentiation following SIVΔnef Vaccination by Transcription Factor Expression Profiling
Source: PLoS Pathog. 2015 Mar 13;11(3):e1004740. doi: 10.1371/journal.ppat.1004740 (PMC4358830; doi:10.1371/journal.ppat.1004740)
Supplement: S1 Table — The qPCR primer/probe sets (TaqMan assays) used to quantify mRNA levels of the indicated target transcripts. (PDF) [file ppat.1004740.s001.pdf]

S1 Table. TaqMan assays

Target transcript	ABI TaqMan probe ID
AHR	Rh02839281_m1
BATF	Rh02860686_m1
BCL6	Rh02839507_m1
BCL11B	Rh01102259_m1
BCOR	Hs00372378_m1
PRDM1	Rh02837839_m1
EOMES	Rh01015627_m1
ID2	Rh02796147_m1
*RORC**	custom
RORA	Rh00931148_m1
PBX3	Rh00608415_m1
NFIL3	Rh02915951_s1
IRF4	Rh02850392_m1
RUNX3	Rh02929319_m1
TBX21	Rh02621772_m1
TCF7	Rh02844810_m1
LEF1	Rh01553173_m1
GATA3	Rh02830714_m1

* a custom assay was designed to specifically target *RORC* transcript variant 2 which encodes Rorγt (Reeves RK, et al., Blood (2011) 118: 3321-3330).

The primer and probe sequences are TGAGAAGGACAGGGAGCCAA (Forward), CCACAGATTTTGCAAGGGATCA (Reverse), TCATGAGAACACAAATTGA (probe.)
